# An Evaluation of Nuclei Preparation of the Dormant Axillary Bud of Grapevine for Cell Cycle Analysis by Flow Cytometry

**DOI:** 10.3389/fpls.2022.834977

**Published:** 2022-02-24

**Authors:** Dina Hermawaty, John A. Considine, Michael J. Considine

**Affiliations:** ^1^UWA School of Agriculture and Environment, The UWA Institute of Agriculture, The University of Western Australia, Perth, WA, Australia; ^2^ARC Centre of Excellence in Plant Energy Biology, School of Molecular Sciences, The University of Western Australia, Perth, WA, Australia

**Keywords:** flow cytometry, dormancy, cell cycle, mitotic index, grapevine buds

## Abstract

Whether the division of cells of a dormant meristem may be arrested, e.g., in the G1 phase, has proven to be an extremely difficult hypothesis to test. This is particularly so for woody perennial buds, where dormant and quiescent states are diffuse, and the organ may remain visibly unchanged for 6–9 months of the year. Flow cytometry (FCM) has been widely applied in plant studies to determine the genome size and endopolyploidy. In this study, we present the application of FCM to measure the cell cycle status in mature dormant buds of grapevine (*Vitis vinifera* cv. Cabernet Sauvignon), which represent a technically recalcitrant structure. This protocol illustrates the optimisation and validation of FCM data analysis to calculate the cell cycle status, or mitotic index, of dormant grapevine buds. We have shown how contamination with debris can be experimentally managed and give reference to the more malleable tomato leaves. We have also given a clear illustration of the primary pitfalls of data analysis to avoid artefacts or false results. Data acquisition and analysis strategies are detailed and can be readily applied to analyse FCM data from other recalcitrant plant samples.

## Introduction

Plant growth and development result from a combination of cell division and cell expansion. The meristems are the origin of cell division, supplying naïve cells that differentiate in files through further divisions to establish various plant tissues. Nevertheless, meristematic activity is discontinuous, particularly in organs that undergo a dormant phase, such as seeds of certain species and the proleptic buds of many temperate perennial plants. In such organs, tight regulation between active growth and arrest is a crucial survival strategy, protecting the meristem from unfavourable seasonal conditions (Anderson et al., 2010). Several studies have implicated the regulation of cell division through cell cycle machinery complexes, i.e., the cyclins (CYCs) and CYC-dependent kinase (CDKs), during seed germination and dormancy transition in perennial buds (Hansen et al., 1999; Horvath et al., 2003; Barrôco et al., 2005; Saito et al., 2015; Cembrowska-Lech and Kêpczyński, 2016). However, few studies have demonstrated discrete regulation at a physiological level especially in perennial buds (Owens and Molder, 1973; Velappan et al., 2017; Table 1). The DNA content (size) is different at each cell cycle phase due to the DNA replication process. Therefore, mitotic activity can be investigated by measuring the nuclear DNA content of the cell. Microscopy and microspectrophotometry with Feulgen staining techniques were widely used to estimate plant nuclei genome size (DNA content) (McLeish and Sunderland, 1961; Price, 1988). Although the Feulgen microdensitometry is a reliable and accurate tool for measuring DNA content, sample preparation is laborious, data acquisition is slow, and the DNA content data are usually generated by averaging a very limited number of nuclei (Michaelson et al., 1991).

**TABLE 1:** Assessment of the quality of nuclei suspension prepared from grapevine buds and young tomato leaves.

Tissue	CV-G1	CV-G2	Debris factor
Grapevine buds	12.98 ± 2.364b	13.11 ± 2.357b	62.86 ± 5.928b
Tomato leaves	4.42 ± 0.820a	4.47 ± 0.808a	32.27 ± 5.752a

*Nuclei quality is represented by the coefficient of variation (CV) of G1 and G2 peak of propidium iodide (PI) histogram generated by FlowJo software. All values are shown in percentage. Means followed by different letters indicate significant differences at p < 0.05 according to Tukey’s multiple comparison test (n = 4).*

Flow cytometry (FCM) has been increasingly used as an alternative, which is able to provide a rapid automated analysis of a large number of nuclei and to generate a more robust measurement of DNA content in plant nuclei (Galbraith et al., 1983; Michaelson et al., 1991; Doležel et al., 2007; Loureiro and Castro, 2015). In combination with DNA staining, FCM is a powerful tool that enables the estimation of genome size, determination of ploidy, and analysis of cell cycle (Doležel et al., 2007). In principle, a nuclei suspension is stained with a DNA-specific fluorescent dye and passed through a fluidic stream to create a single file of nuclei, allowing individual analysis of the nuclei. The excitation of the fluorophore by the light source results in light scattering, which is collected *via* optics and directed through a series of filters and dichroic mirrors, detected by photomultiplier tubes (PMTs), and digitised, providing quantitative data that can reveal the physical properties of the fluorescing object (Picot et al., 2012). However, the interpretation of the FCM data requires a robust understanding of the composition of the suspension, particularly in tissues that are recalcitrant for the isolation of nuclei or have contamination with autofluorescent compounds.

The establishment of the razor chopping method of plant tissue in nuclei isolation buffer to release nuclei into suspension provides a rapid and convenient approach to evaluate the precision and accuracy of the FCM data from plant cells. This technique enables the sources of error to be resolved, e.g., irregular shape or size and is less time-consuming than the preparation of protoplasts (Galbraith et al., 1983). Despite the convenient and universal application of razor chopping for preparing nuclei suspensions, at present, there is no single nuclei isolation buffer that successfully resolves nuclei from all plant tissues. Ideally, a suitable nuclei isolation buffer could provide buffering capacity to maintain pH suitable for fluorescence dye activity (Loureiro et al., 2006a) and minimise nuclei degradation and contamination. However, plant tissues are commonly rich in secondary metabolites which interfere with nuclear isolation and fluorescent detection. The presence of secondary metabolites, such as caffeine, chlorogenic acid (polyphenols precursor), and tannic acid, has been previously reported to cause a stoichiometric error by either increasing or decreasing the fluorescence signal of propidium iodide (PI), a nucleic acid dye (Noirot et al., 2000; Price et al., 2000; Loureiro et al., 2006b). Noirot et al. (2000) noted that dilution with isolation buffer or nuclei isolation by centrifugation could reduce the negative effect of contamination by secondary but could not eliminate the effect entirely. Moreover, Price et al. (2000) suggested that caution must be taken when interpreting samples with secondary metabolite contaminations.

The fluorescing debris may still prevail within nuclei suspensions prepared from plant tissues even after optimising the nuclei preparation procedure, thus requiring careful data analysis and interpretation. For example, the presence of debris in the nuclei suspension and accessibility of DNA-specific dye can be judged by analysing histograms of the relative fluorescence signal, while the coefficient of variation (CV) value of the histogram peaks reflects the precision of the FCM data. Fluorescence and light scatter data collected by the FCM instrument enable an *in silico* (gating) extraction of pure and intact nuclei and can be used to increase the accuracy of analysis using FCM (Doležel et al., 2007). Furthermore, an epifluorescence microscope can then be used to examine nuclear integrity by observing the appearance of debris-free vs. debris-contaminated nuclei (Marie and Brown, 1993).

In this experiment, we sought to evaluate the nuclear preparations to properly interpret the FCM data, using nuclear preparations of tomato leaves as an internal standard. A preliminary study has been conducted to select the suitable nuclei isolation buffer for grapevine buds, i.e., MgSO_4_ buffer (data not shown), which is also in agreement with the previous report to be the most suitable buffer for a sample with high tannin or phenolic compounds (Wang et al., 2015). This protocol will focus on data acquisition strategies and data analysis to identify artefacts that could lead to inaccurate and false results.

## Materials and Equipment

### Materials

Mature, dormant buds of grapevine (*Vitis vinifera* cv. Cabernet Sauvignon) were harvested from vines within a commercial cane-pruned vineyard in the temperate/Mediterranean region of Margaret River, Western Australia (34°S, 115°E). Canes were cut from the vines and trimmed to five nodes from the 4th to the 8th node (1st clear node should be ≥10 mm from the base of the branch). The canes were immediately transported to the laboratory in damp newsprint in an insulated box and stored at 22°C for 24 h. The cuttings were then stored in the dark for 24 h before processing and subsequent analysis. Tomato (*Lycopersicum esculentum* cv. Money Maker) seeds were germinated and grown in vermiculite. Seedlings were watered daily and maintained at 22°C under cool white LED light and a 12-h photoperiod. For the FCM analysis, young leaves were harvested from seedlings with at least four leaves emerging.

### Reagents

• Magnesium sulphate heptahydrate, MgSO_4_.7H_2_O (Sigma-Aldrich, Cat. no. M2773).
• Potassium chloride, KCl (Sigma-Aldrich, Cat. no. P5405).
• HEPES (Sigma-Aldrich, Cat. no. H3375).
• Dithiothreitol (DTT; Sigma-Aldrich, Cat. no. DDT-RO 10708984001).
• Triton X-100 (Sigma-Aldrich, Cat. no. T9284).
• Polyvinylpyrrolidone (PVP) 40 (Sigma-Aldrich, Cat. no. PVP40).
• Ribonuclease A (Sigma-Aldrich, Cat. no. R6513).
• Propidium iodide (Sigma-Aldrich, Cat. no. P4170).

### Equipment

• 2-ml clear centrifuge tube.
• 2-ml amber centrifuge tube.
• 50-ml conical tube.
• 40-μm nylon filter (or Corning blue cell strainer, Cat. No. CLS431750).
• 100-μm nylon filter (or Corning yellow cell strainer, Cat. No. CLS431752).
• 5-ml Round Bottom Polystyrene FACS Tubes.
• Cooler box.
• Disposable Petri dish (10-cm diameter).
• Disposable transfer pipette (2 ml).
• Fine forceps.
• Fridge (4°C) and freezer (−20°C).
• Light and fluorescence microscope with 20× and 40× Plan-Neofluar objectives, UV or blue epi-illumination, and differential interference contrast filters.
• Magnetic stirrer.
• Micropipettes and corresponding tips (10–1,000 μl).
• Microscope glass slides and coverslips.
• pH meter.
• Refrigerated centrifuge.
• Vortex mixer.

### Reagent Set-Up

• Nuclear isolation buffer (100 ml): 0.246 g MgSO_4_.7H_2_O, 0.3727 g KCl, 0.1191 g HEPES, 0.1 g DTT + 90 ml MilliQ water. pH was checked and adjusted to 7.3–7.4. Of note, 500 μl Triton X-100 was added and topped up to 100 ml with MilliQ water, mixed well with a magnetic stirrer, made up to 10 ml aliquots, and stored at −20°C. Notably, 3 g PVP-40 was added just before use.
• DNAse-free RNase (1 mg ml^–1^): 10 mg Ribonuclease A + 10 ml MilliQ water. The solution was gently vortexed for 60 s and incubated at a 90°C water bath for 20 min with occasional shaking. It was then cooled at room temperature and stored in 500 μl aliquots at −20°C.
• PI stock solution (1 mg ml^–1^): 10 mg PI + 10 ml MilliQ water. The solution was mixed well by vortexing. Of note, 100 μl aliquots were transferred to several amber tubes and stored at 4°C for storage up to 6 months or at −20°C for longer storage.

## Methods

### Nuclei Suspension Preparation (Timing ∼1–2 h)

1. Notably, 50 mg (∼10 buds) of the sample was chopped finely with razor blades on a Petri dish containing 500 μl cold NIB. A mixed sample was prepared by co-chopping 50 mg of buds with 25 mg of tomato leaves.
2. Another 2.5 ml NIB (500 μl at a time) was added while washing the Petri dish to collect all fragments.
3. The sample was incubated on ice for 1 h and then filtered through a 100-μm nylon mesh filter one time and a 40-μm filter two times.
4. The filtrate was transferred into a new 2-ml tube.
5. The nuclei were spun down at 2,000 rpm for 8 min at 4°C.
6. Of note, 1,000 μl new NIB was then added to a decant supernatant.
7. Notably, 500 μl of nuclei suspension was transferred to a new 2-ml amber tube.
8. To the rest of the nuclei suspension in the clear 2-ml tube, 500 μl NIB was added.

*Note: This is the unstained sample. It can be kept in a clear tube.*

9. To the nuclei suspension in the amber tube, 430 μl NIB, 50 μl PI stock solution (final concentration of 50 μg/ml), and 20 μl RNase stock solution (final concentration of 20 μg/ml) were added and kept on ice until analysis.

*Note: This is the stained sample, which must be kept in an amber tube all the time.*

### Flow Cytometry Data Acquisition and Analyses (Timing 10–30 Min per Sample)

The nuclear DNA content stained with PI can be measured using an FCM instrument equipped with blue laser light (488 nm) and a red fluorescence light detector. In this study, data acquisition was conducted using the BD FACS Canto™ II (BD Biosciences, San Jose, CA, United States) cytometer equipped with an air-cooled 488-nm solid-state 20 mW laser, and fluorescent signal was collected through a 556-dichroic long-pass filter and a 585/42-nm band-pass filter. Nuclei suspensions were analysed in a single tube acquisition mode. Data were analysed with BD FACSDiva™ (BD Biosciences) and FlowJo (Tree Star Inc., Ashland, OR, United States) software.

10. In the global worksheet window, the following visualisation plots were created:
  a. Forward scatter vs. side scatter (FSC vs. SSC) dot plot to visualise all particles detected in the nuclei suspension and differentiate clump, doublet, and debris.
  b. PI-DNA fluorescence vs. FSC (PI-DNA vs. FSC) dot plot to isolate the PI-positive events from background noise.
  c. PI-DNA histogram to identify G1 and G2 peaks.
11. At the beginning of each FCM session, the PMT voltage was adjusted, the initial gate was created, and the acquisition parameters were set as the following.

#### Adjusting Photomultiplier Tube Voltage (5–10 Min)

12. Stained samples were run in the flow cytometer, and the dots (events) that were visualised inside the FSC vs. SSC plot were observed to adjust PMT voltages. At this stage, no data recording is needed.
13. The PMT voltages were adjusted to maintain a good dot dispersion, and that dots were not condensed up against the top and right border (highest value) of the axis and then stopped running.
14. Using the same PMT voltage setting, the negative control sample was run, and a similar observation with the stained sample was repeated, i.e., dots were not condensed up against the bottom and left border (the lowest value) of the axis.
15. The axis of the FSC vs. SSC plot was changed into PI-DNA (*X*-axis) and FSC (*Y*-axis); a total of 15,000 events were run and recorded and then stopped running.
16. The dispersion of the dots of the recorded events was observed, and a rectangular gate was created at the area where dots were relatively low.

*Note: Observation using the PI-DNA vs. FSC aims to differentiate between stained nuclei and background noise or autofluorescence particles. All events (dots) inside the rectangular gates are particles that only emit light if PI is bound to it, while the dots outside this gate are background noise that emits fluorescence light regardless of the presence of PI in its particle (autofluorescence particle).*

17. The stained sample was run, and the dispersion of the dots was observed in the PI-DNA vs. FSC plot. A dense nuclei cluster should be visible at this stage.
18. The PMT voltage was readjusted again so that the most distinct nuclei cluster was located at around channel 50–100 K.
19. If PI-positive nuclei clusters are not well defined in the PI-DNA vs. FSC plot, the PI-DNA histogram is used. The histogram will show the most prominent nuclei cluster as peak(s). Again, the PMT voltage was readjusted so that the most distinct peak was located at around channel 50–100 K.

*Note: It is common to find a peak that belongs to autofluorescence debris. The peak is usually located at a channel lower than 50 K. Autofluorescence debris can be discerned from the nuclei population by increasing the PMT voltage gradually. If the position of the peak is not shifting, it is the debris. If the peaks shift after PMT voltage is increased, it is the nuclei.*

20. The instrument is now ready to use. The same voltage setting was kept throughout the data acquisition process. These set-up steps must be conducted at the beginning of the FCM session, i.e., after turning on the FCM instrument.

#### Data Acquisition (Timing 10–30 Min per Sample)

21. In the acquisition dashboard in BD FACSDiva™ software, the fluid flow was set to low, storing gate to all events, and stopping gate to 15,000 of PI-positive events.

*Note: Always the data of all events are stored, as this will allow further analysis using third-party FCM software*. *The stopping gate is set at a particular value (15,000 events in this protocol) to ensure that the cell cycle or mitotic index analysis is conducted using a similar number of events (nuclei population).*

22. The stained sample was run until the stopping gate cut-off value was achieved. Then, the negative control was run.

*Note: Negative control sample will never achieve the number of events set for the stopping gate, and therefore, it is best to keep the same amount of all events as recorded in the stained sample (or a sample using the same acquisition time is run)*. *This way, the gate of the positive event (step 12) is made by comparing the same amount of events between stained and unstained samples.*

23. Each run data was saved in an FCS file.

#### Data Analysis

24. The FCS files that were saved from the acquisition step were opened using the FCM analysis software. In this protocol, we used the FlowJo V10 (Tree Star Inc., Ashland, OR, United States). The Watson pragmatic algorithm is used to model the cell cycle and calculate the proportion of each cell cycle phase and the CV.

*Note: Several FCM software analyses are available for free, e.g., Flowing Software by University of Turku^1^ or the web-based analysis tools by the Floreada Cytometry team^2^*.

25. The quality of the histogram and nuclei preparation procedure is evaluated by the CV value, background debris factor (DF), and percentage of intact nuclei. The CV value was generated from the Watson pragmatic algorithm. DF was calculated according to the study by Loureiro et al. (2006a) as follows:

$$D\,F=\frac{\Sigma \,P\,I\,p\,o\,s\,i\,t\,i\,v\,e\,p\,a\,r\,t\,i\,c\,l\,e\,s-\Sigma \,i\,n\,t\,a\,c\,t\,n\,u\,c\,l\,e\,i}{\Sigma \,P\,I\,p\,o\,s\,i\,t\,i\,v\,e\,p\,a\,r\,t\,i\,c\,l\,e}\times 100$$

where “intact nuclei” is a population of PI-positive particles having relatively similar size (FSC-A) and optical complexity (SSC-A). This population is obtained after applying *in silico* gates, as shown in Figures 2D,I.

## Results

For simplification, the term dot plot will be used for the biparametric plot and histogram for the uniparametric histogram. The FSC is light that was collected at a relatively forward direction to the light source (usually a laser beam), which can estimate the relative size. The SSCs refer to light refracted to all directions mainly caused by the particle’s internal structure, meaning particles with fewer internal cellular structures will produce fewer SSC lights. Our result also observed an association between particle topology with SSC data, i.e., a suspension containing debris-coated nuclei shows an incident of high SSC value. Combining FSC and SSC data will enable us to identify particles of interest based on the size and topology of the particle; in our case, it is a nuclei population that has relatively a similar size and is free from debris contamination (fewer SSC lights). In addition to the direction of the light diffusion, FCM also expressed light data as height, width, and area. The width value corresponds to the time spent for a particle travelling across the laser beam. A larger particle travels longer than a smaller particle; therefore, the width value will be higher and proportional to particle size. The height value corresponds to the intensity of emitted fluorescence; in the measurement of nuclear DNA content, a bigger genome binds more DNA stain, thus higher fluorescence intensity. Finally, area (A) indicates the value of light intensity relative to the size of the particle, and we used this value in this study.

**FIGURE 1:**
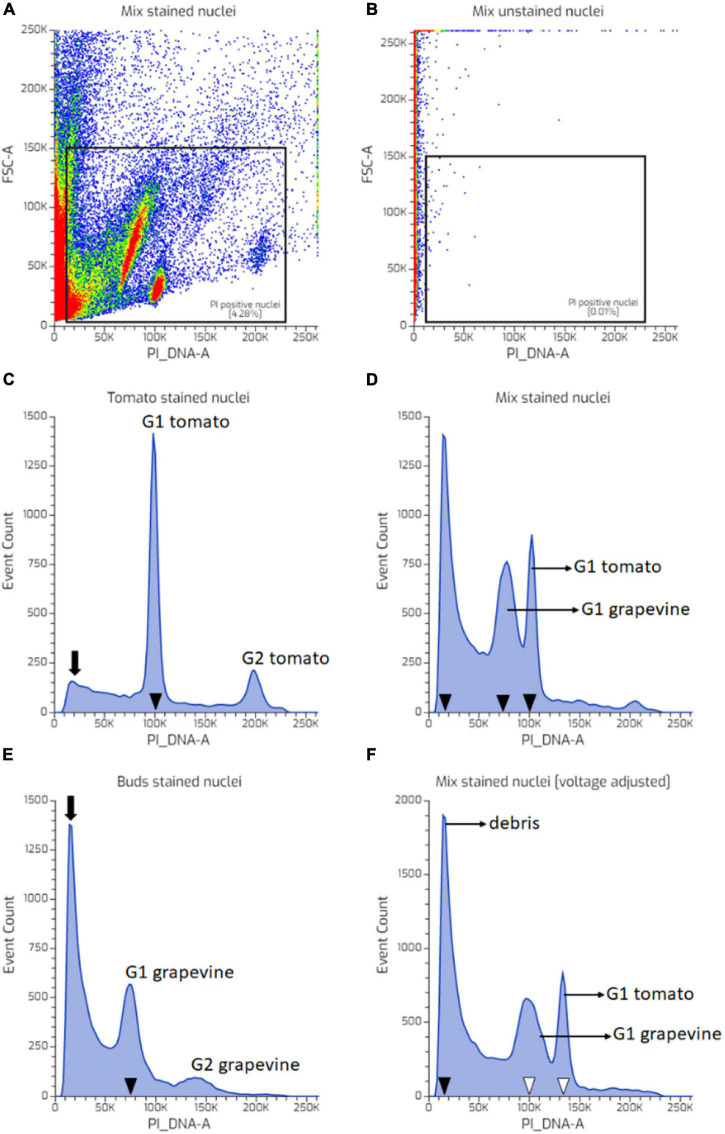
Flow cytometry (FCM) instrument setting and identification of grapevine bud nuclei peak, using tomato nuclei as an internal standard. **(A)** A uniparametric distribution of propidium iodide (PI)-stained mix nuclei suspension with one of the highly abundant populations (red coloured) adjusted to be in the channel 100 K. **(B)** A uniparametric distribution of unstained mix nuclei suspension. **(C)** A uniparametric distribution of PI-stained tomato nuclei suspension analysed using the same instrument setting as **(A)** showing G1 peak located in the channel 100 K. **(D)** A uniparametric distribution of PI-stained mixed nuclei suspension sample showing three major peaks, i.e., at channel 100 K belong to tomato G1 nuclei (as shown in **C**), at 75 and 20 K (full arrow heads). **(E)** A uniparametric distribution of PI-stained grapevine nuclei suspension analysed using the same instrument setting as **(A,D)** showing G1 peak located in the channel 75 K. **(F)** Increasing sensitivity of the PI_DNA photomultiplier tube (PMT) voltage resulted in shifting the G1 peaks of the grapevine and tomato nuclei to a higher PI_DNA fluorescence channel (empty arrow heads), while particles located at channel 20 K (the third peak shown in **D**) remained at the same position (arrows) showing that these particles are debris which originating from grapevine nuclei suspension. Colors in dot plot indicate nuclei population density, being red as the highest and blue the lowest.

Young tomato leaves were used as an exemplar tissue to investigate the instrument settings. Figure 1 illustrates the representative dot plot and histogram distributions of nuclei prepared from tomato and a mix of tomato leaves and grapevine buds. The PI-positive population was gated relative to the unstained and stained sample (Figures 1A,B). Two peaks of the tomato nuclei separated well and were identified as G1 and G2 peaks (Figure 1C). To identify the grapevine G1 peak, the PI_DNA PMT voltage was adjusted to position the tomato G1 peak at channel 100 K (Figure 1C, filled arrowhead). A mixed suspension of tomato and grapevine nuclei from a co-chopped sample was then run with this instrument setting. Three major peaks were observed (Figure 1D). The peak located at around channel 100 K was identified as the tomato G1 peak, and the peak located between channel 50 and 100 K was identified as the grapevine G1 peak. The third peak located at the PI_DNA channel less than 50 K was detected only in the co-chopped sample and originating from the grapevine bud sample (Figure 1E, arrow). Using the data illustrated in Figure 1F, we confirmed that the third peak consists of weakly fluorescing debris as these objects did not correlate with the PI_DNA fluorescence channel. The G1 peak shifted to a higher channel when PI_DNA PMT voltage was increased (Figure 1F, empty arrowheads), and yet, the debris remained in the same fluorescence channel (Figure 1F, filled arrowhead). The presence of debris in the co-chopped sample did not affect the position of both tomato and bud G1 peak (Figures 1C–F).

**FIGURE 2:**
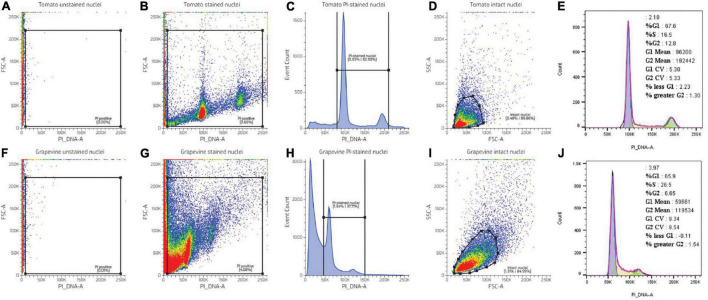
FCM data processing illustrated using nuclei suspension prepared from tomato **(A–E)** and grapevine buds **(F–J)**. PI-positive events were extracted by creating a rectangular gate in the forward scatter area (FSC)-A vs. PI_DNA-A dot plot **(B,H)**. The “PI-positive” gate was positioned so that it excluded the background noise observed in the unstained sample **(A,F)**. Weakly fluorescing debris at lower PI_DNA channels was excluded by creating a gate between the PI-stained nuclei population **(C,H)**. Debris-coated nuclei were excluded by plotting the “PI-stained nuclei” into the FCS-A vs. side scatter area (SSC)-A dot plot and creating a polygonal gate around the intact nuclei population **(D,I)**. The proportion of nuclei in G1 and G2 phases was modelled using a Watson pragmatic algorithm (Watson et al., 1987) from the intact nuclei population **(E,J)**. Colors in dot plot indicate nuclei population density, being red as the highest and blue the lowest.

**FIGURE 3:**
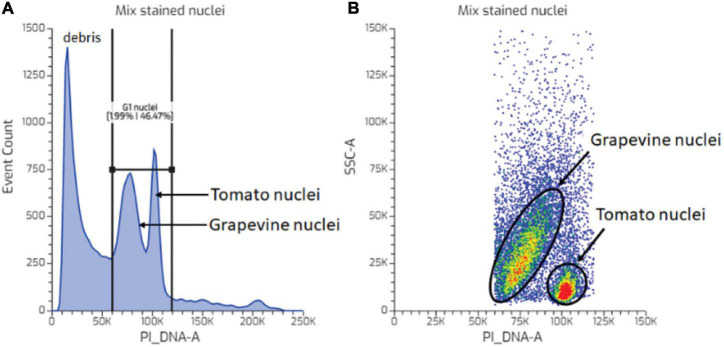
Nuclei complexity analysis. **(A)** PI-stained events of a mixed nuclei suspension sample of grapevine bud and tomato leaf. **(B)** Optical complexity of tomato and grapevine bud nuclei from the gated PI nuclei events in **(A)**. Colors in dot plot indicate nuclei population density, being red as the highest and blue the lowest.

Further evaluation showed that despite the identical nuclei preparation, the tomato and grape nuclei showed a different FCM profile. Two well-separated nuclei populations were observed in the tomato dot plot of the FSC area vs. the PI_DNA fluorescence area (FSC-A vs. PI_DNA-A), relative to the unstained nuclei (Figures 2A,F), which resulted in two separated peaks in the histogram, representing G1 and G2 nuclei (Figures 2B,C). This was not the case with grapevine buds, where the nuclei populations appeared contiguous with the background debris (Figure 2G). As such, identifying the G1 and G2 peaks of the grapevine nuclei was only possible through the histogram (Figure 2H). This result was also reflected in the difference in the CV value (Figures 2E,J), with grapevine buds having a higher G1 peak CV of 12% compared to tomato, i.e., 4% (Table 1). The visualisation of the PI-stained nuclei using an SSC-A vs. PI_DNA-A dot plot showed the difference between the optical complexity profiles of the nuclei suspensions. It was found that nuclei prepared from grapevine buds were more optically complex than nuclei prepared from tomato leaves, indicated by the broader range of the SSC-A value of grapevine compared to tomato (Figures 2D,I). The optical complexity observation in the co-chopped sample also showed a similar pattern (Figure 3).

**FIGURE 4:**
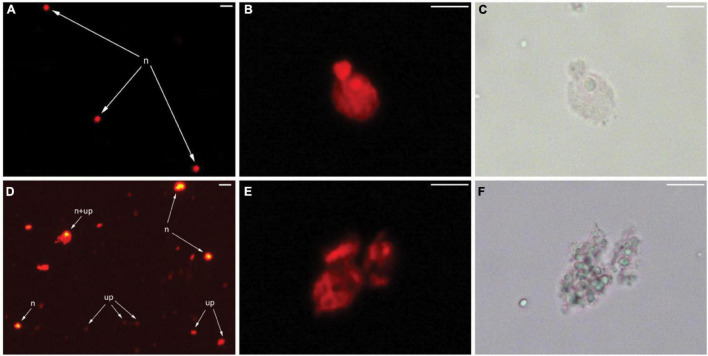
Micrographs of tomato leaf and grapevine bud nuclei suspensions under light and epiluminescence microscope. A fluorescence image of debris-free nuclei (n) prepared from tomato young leaves **(A)**. Higher magnification of a single nucleus of tomato by fluorescence **(B)** and bright-field **(C)** illumination. Overexposed fluorescence image of grapevine bud nuclei suspension showing debris particles with weak fluorescence (up), debris aggregated with nuclei (n + up), and debris-free nuclei (n) **(D)**. Higher magnification of a single nucleus of grapevine by fluorescence **(E)** and bright-field **(F)** illumination. Bar = 20 μm **(A,D)**, 10 μm **(B,C,E,F)**.

The microscopic observation was then carried out to inspect the morphology of the nuclei and debris particles in the nuclei suspension prepared from grapevine buds. An overexposed fluorescence examination confirmed numerous unknown particles with a weak fluorescence signal (Figure 4D) found in nuclei suspension prepared from the grapevine but comparatively absent from the nuclei suspension of tomato (Figure 4A). An aggregate of this unknown particle was attached to the nuclei and interfered with the fluorescence signal (Figure 4D). The examination of the nuclei at a higher magnification of 400× showed an irregular shape of the grapevine nuclei (Figures 4E,F), compared to the round/elliptical shape of tomato nuclei (Figures 4B,C).

## Discussion

Tissues of woody perennials are known to be recalcitrant for nuclei preparation and FCM analysis mainly due to the presence of undesirable cytosolic compounds (Loureiro et al., 2007). Noirot et al. (2000) noted an interaction between the effects of cytosol on DNA-dye binding, resulting in variation in the estimation of the nuclear DNA content. Furthermore, it was suggested to use an internal standard that exhibits similar sensitivity to DNA-dye accessibility to target nuclei. Tomato was chosen as a suitable candidate for grapevine bud nuclear DNA measurement for two reasons: (1) tomato genome size is almost two times that of thus the G1 peak will not overlap and well separated from each other grapevine (Arumuganathan and Earle, 1991; Lodhi et al., 1994; Leal et al., 2006); (2) cytosolic compounds were also suggested to be present in tomato nuclei suspension thus expected to have a similar DNA-dye accessibility (Price et al., 2000). The latter is more crucial in an experiment that aims to measure changes in the proportion of G1 and G2 nuclei during dormancy, and hence, the high-quality fluorescence data are essential. An event such as endoreduplication, which is common in tomato tissue, may result in the variation of the genome size (ploidy level), but this will be more problematic in an experiment aiming for measuring genome size. Regarding the quality assessment of sample preparation, Loureiro et al. (2006a) suggested using CV and DF values. The CV value indicates the integrity of nuclei and the variation of the fluorescence signal, and the DF value assesses the quality of the nuclei suspension preparation by calculating the proportion of high-quality intact nuclei in the nuclei suspension. A good nuclei suspension preparation is expected to contain a high proportion of intact nuclei compared to debris particles.

The results showed that the tomato tissue we used in this experiment fulfilled the criteria as an excellent internal standard, i.e., the G1 peak of both tomato and grapevine buds was well separated, and the position of the tomato PI fluorescence channel remained unchanged in the co-chopped sample. The latter suggested that the DNA-dye accessibility remained the same after exposure to the cytoplasmic content of grapevine buds and thus suggested that it was unlikely that the presence of cytosolic compounds from grapevine buds was acting as a barrier for PI binding (Price et al., 2000). Nevertheless, the peak attributes of nuclei from these species differed, with tomatoes having a considerably smaller CV value than grapevine buds in individual and co-chopped samples (Table 1). This indicates that inhibitors reduced the quality of intact nuclei of grapevine buds rather than interfering with PI binding to DNA.

In FCM, FSC is light that scatters in the same direction as laser beam (light source), and the value of FSC-A is indicative of the relative size of a particle. The light scattered at a 90° angle relative to the incident beam is referred to SSC, and the SSC-A value represents the degree of optical complexity of the particle, for example, granularity within the sample and surface irregularity (Jaroszeski and Radcliff, 1999; Picot et al., 2012). In clinical and medical research, the SSC-A value is used to identify and separate the components of white blood cells (Ruzicka et al., 2001; Tarrant, 2005). For example, granulated cells, such as neutrophils and eosinophils, tend to have a higher SSC-A because the granule inside the cells scatters more lights than lymphocytes, for instance, which is ungranulated. Using the same principle, we used the SSC-A vs. PI_DNA-A plot to evaluate the optical complexity of PI-stained nuclei gated from the PI_DNA-A histogram (Figure 3). The PI-stained nuclei of grapevine buds showed more variable optical complexity compared to the more uniform tomato nuclei. This was indicated by a broader range of SSC-A values in grapevine bud nuclei compared to tomato nuclei. A reasonable amount of particles with high optical complexity was also observed from the FSC-A vs. SSC-A plot, i.e., the blue dot outside the “intact nuclei gate,” and we suggest that these were nuclei coated with debris (Figure 4). The ratio of these high optical complex particles over debris-free intact nuclei is the background DF percentage presented in Table 1. Microphotographs of nuclei suspension stained with PI under epiluminescence complement the data from the FCM analysis. The tomato nuclei suspension showed a very low amount of fluorescing debris, which resulted in cleaner and more debris-free nuclei, and thus more uniform SSC-A and FSC-A values (Figure 2D). On the contrary, the grapevine bud nuclei suspensions showed a similar profile to the nuclei treated with tannic acid, reported by Loureiro et al. (2006b). A high amount of weakly fluorescent debris was found as aggregates of the debris and aggregates attached to the nuclei, which may explain the debris with a very high SSC-A value and the variable optical complexity of grapevine bud nuclei, respectively (Figure 2I). The routine application of FCM in animal cells limits the interpretation of SSC data to granularity, such as when dealing with blood cells. In this study, we represented the possibility of using SSC data to help determine nuclei quality prepared from plant tissue and incorporate it in the analysis to produce a more accurate data output.

## Conclusion

This study provides an FCM protocol for examining cell cycle status in mature dormant grapevine buds. We fine-tuned data acquisition and analysis based on the previously published methods for estimating genome size in plants. In this protocol, we incorporated the use of the internal standard for instrument calibration, post *in silico* extraction (gating) to exclude background noise further, and the examination of nuclei integrity using epiluminescence microscopy. Further optimisation can be included in the protocol by emphasising the selection of isolation buffers for nuclei suspension preparations as described elsewhere. We believe this protocol will allow reproducible data acquisition, analysis, and interpretation, especially in the FCM analysis of recalcitrant tissue.

## Data Availability Statement

The raw data supporting the conclusions of this article will be made available by the authors, without undue reservation.

## Author Contributions

MC conceived and supervised the project. DH planned, performed all the experiments, analyzed the data, prepared all the figures, and wrote the manuscript with constructive comments from MC. JC performed the optimization of the FCM procedure and microscopy analysis. All authors contributed to the article and approved the submitted version.

## Conflict of Interest

The authors declare that the research was conducted in the absence of any commercial or financial relationships that could be construed as a potential conflict of interest.

## Publisher’s Note

All claims expressed in this article are solely those of the authors and do not necessarily represent those of their affiliated organizations, or those of the publisher, the editors and the reviewers. Any product that may be evaluated in this article, or claim that may be made by its manufacturer, is not guaranteed or endorsed by the publisher.

## Footnotes

1
https://bioscience.fi/services/cell-imaging/flowing-software/

2
https://floreada.io/
